# Supplementary material for: Evaluation of the insecticide custody chain and its relationship with malaria burden in the Brazilian Amazon: a process and exploratory impact assessment (2019-2023)
Source: Mem Inst Oswaldo Cruz. 2026 Mar 6;121:e250152. doi: 10.1590/0074-02760250152 (PMC12965720; doi:10.1590/0074-02760250152)
Supplement: Supplementary material [file 1678-8060-mioc-121-e250152-s1.pdf]

TABLE I

Description of the continuous explanatory variables used in the statistical modeling of the study

Variable	Type	Observations
Malaria cases	Continuous	Absolute count of autochthonous cases registered per year/municipality

TABLE II

Classification of the dependent variable used in the study's statistical analysis

Variable	Type	Units / Characteristics
Average temperature	Continuous	Degrees Celsius (°C), annual average per municipality/region
Average rainfall	Continuous	Millimeters (mm), average annual rainfall
Dispensing insecticide	Continuous	Annual quantity in kilos (kg) of insecticide Etofenproxi - PM sc 20% distributed
Population density	Continuous	Inhabitants per km² (derived from population base and territorial area)
Deforestation	Continuous	Deforested area per year (in km²), measured by satellite (data from INPE/PRODES)

TABLE III

Description of nationally notified malaria cases from 2019 to 2023

Cases	2019	2020	2021	2022	2023
National	157.459	145.192	140.474	131.224	142.834
Annual percentage change		-7,8%	-3,2%	-6,6%	8,8%

Source: Ministry of Health. Sivep-Malaria.

TABLE IV

Description of data regarding the latest purchases of Etofenproxi - PM 20% insecticide in kilograms, at the federal level in the years 2019 to 2023

Insecticide	2019	2020	2021	2022	2023
Etofenproxi - PM sc 20% (kg)	89.000	60.010	50.000	60.000	24.000
Annual percentage change		-32%	-16%	20%	-60%

Source: Ministry of Health.

TABLE V

Description of the number of kilos dispensed of the insecticide Etofenproxi - PM sc 20% in the years 2019 to 2023

Municipality	2019	2020	2021	2022	2023
São Gabriel da Cachoeira	1120	2280	2840	0	1000
Variação		104%	25%	-100%	100%
Tefé	7080	2920	2000	4200	3100
Variação		-59%	-32%	110%	-26%
Barcelos	1400	2320	2520	2400	480
Variação		66%	9%	-5%	-80%

Source: Amazonas State Health Surveillance Foundation.

TABLE VI

Agglutinated data on insecticide dispensation, national malaria cases, 99% of which are concentrated in the Amazon region, temperature, rainfall, population base and deforestation, from 2019 to 2023

Year	Dispensing insecticide (kg)	National cases	Temperature average (°C)	Rainfall average (mm)	Population	Deforestation (km^2)
2019	89.000	157.459	26.1	284,22	28100000	10129
2020	60.010	145.192	26.2	242,87	28100000	11088
2021	50.000	140.474	25.9	229,34	29796546	13235
2022	60.000	131.224	25.9	181,12	27800000	11568
2023	24.000	142.834	26.4	231,96	29796546	9375

TABLE VII

Average annual temperature and rainfall for the Amazon region from 2019 to 2023

Year	Average temperature (°C)	Average rainfall (mm)
2019	26,1	284,22
2020	26,2	242,87
2021	25,9	229,34
2022	25,9	181,12
2023	26,4	231,96

Source: National Institute of Meteorology - INMET.

TABLE VIII

Deforestation data for the Amazon region from 2019 to 2023

Year	Deforestation (km2)
2019	10129
2020	11088
2021	13235
2022	11568
2023	9375

Source: National Institute for Space Research - INPE, PRODES Project.

TABLE IX

Simple linear regression between environmental variables and malaria cases (Brazil, 2019-2023)

Independent variable	Coefficient (β)	Value -p	95%CI Bottom	95%CI Top	R^2 adjusted
Insecticide (kg)	0,161	0,038	0,015	0,308	0,74
Average temperature (°C)	4,156	0,441	-9,586	17,898	0,00
Average rainfall (mm)	0,228	0,057	-0,009	0,466	0,66
Deforestation (km^2)	0,626	0,034	0,090	1,163	0,76

CI: confidence interval.

TABLE X

Insecticide chain of custody risk matrix for BRI actions to prevent, control and eliminate malaria

Process	Identified risk	Probability	Impact	Mitigation measure
Demand planning and Forecasting	Inaccurate estimates leading to either a shortage or surplus of insecticides.	High	High	Implementation of an integrated data system to improve demand forecasting.
Procurement and Product Quality	Delays in procurement resulting in stockouts.	Medium	High	Optimization of procurement processes to reduce acquisition lead time.
Storage and Transport	Inadequate infrastructure compromising product quality.	High	High	Investment in adequate infrastructure and continuous monitoring of storage conditions.
Distribution and application	Shortage of personnel for malaria control actions and limited logistical resources, resulting in inadequate coverage and application failures.	High	High	Workforce expansion with emergency hiring and training of new staff, along with strengthened logistics to ensure efficient coverage.
Monitoring and evaluation	Deficient monitoring hindering early detection of failures.	Medium	Medium	Development of a digital system for monitoring and evaluating action effectiveness.

BRI: risk matrix for the insecticide chain of custody.

The purpose of this risk matrix is to identify, classify and prioritize the main risks associated with the four operational axes of the chain of custody of the insecticide used in BRI actions: (1) Planning and programming; (2) Storage and distribution; (3) Application and execution in the field; and (4) Monitoring and evaluation.
Each risk identified was assessed based on two criteria: probability of occurrence (low, medium or high) and potential impact on the effectiveness of the vector intervention (low, moderate or high). The combination of these criteria resulted in the overall risk classification, according to the color scale:
- Low risk (low priority for action)
- Moderate risk (requires continuous monitoring)
- High risk (immediate corrective action recommended)

The matrix highlights critical points in the chain, such as failures in predictive planning, lack of integration between information systems (SIVEP, SIES and FVS), poor traceability of inputs, lack of systematic monitoring of vector resistance, and weak federal coordination. These factors increase the risk of shortages, misallocation of resources and a reduction in effective BRI coverage, jeopardizing the achievement of the goals of the National Malaria Elimination Plan (PNEM).

TABLE XI

Description of notified malaria cases from 2019 to 2023

Municipality	2019	2020	2021	2022	2023
Barcelos	8794	7185	10457	6662	6986
Variação		-18%	46%	-36%	5%
São Gabriel da Cachoeira	8597	10118	10092	9075	8383
Variação		18%	0,3%	-10%	-8%
Tefé	2590	1581	3374	1478	2844
Variação		-39%	113%	-56%	25%

Source: Ministry of Health. Epidemiological Surveillance Information System (Sivep-Malaria), 2022.

TABLE XII

Average annual temperature and rainfall for the municipalities of Barcelos, Tefé and São Gabriel da Cachoeira from 2019 to 2023

Year	Barcelos temperature average (°C)	Barcelos average rainfall (mm)	Tefé temperature average (°C)	Tefé average rainfall (mm)	São Gabriel da Cachoeira temperature average **(°C)**	São Gabriel da Cachoeira average rainfall (mm)
2019	26.1	118.0	26.2	123.2	26.0	113.0
2020	26.3	123.1	26.4	128.2	26.2	118.1
2021	25.9	132.9	26.0	138.0	25.8	127.9
2022	25.8	112.9	25.9	117.9	25.7	107.8
2023	26.4	108.2	26.5	113.2	26.3	103.1

Source: National Institute of Meteorology - INMET.

Table XIII

Agglutinated data on insecticide dispensing, malaria cases, temperature, rainfall, population base and deforestation, from 2019 to 2023 for the municipalities of Barcelos, Tefé and São Gabriel da Cachoeira in the state of Amazonas

Barcelos						
Year	Dispensing insecticide (kg)	Malaria cases	Temperature average (°C)	Rainfall average (mm)	Population	Deforestation (km^2)
2019	1.400	8.794	26.1	118.0	25.718	11,05
2020	2.320	7.185	26.3	123.1	25.718	1,26
2021	2.520	10.457	25.9	132.9	25.718	1,39
2022	2.400	6.662	25.8	112.9	18.834	0,71
2023	480	6.986	26.4	108.2	18.834	1,68
Tefé						
Year	Dispensing insecticide (kg)	Malaria cases	Temperature average (°C)	Rainfall average (mm)	Population	Deforestation (km^2)
2019	7.080	2.590	26.2	123.2	61.453	7,54
2020	2.920	1.581	26.4	128.2	61.453	10,47
2021	2.000	3.374	26.0	138.0	61.453	12,78
2022	4.200	1.478	25.9	117.9	73.669	2,23
2023	3.100	2.844	26.5	113.2	73.669	12,39
São Gabriel da Cachoeira						
Year	Dispensing insecticide (kg)	Malaria cases	Temperature average (°C)	Rainfall average (mm)	Population	Deforestation (km^2)
2019	1.120	8.597	26.0	113.0	46.303	6,33
2020	2.280	10.118	26.2	118.1	46.303	6,33
2021	2.840	10.092	25.8	127.9	46.303	5,59
2022	0	9.075	25.7	107.8	51.795	2,4
2023	1.000	8.383	26.3	103.1	51.795	2,99

TABLE XIV

Results of the simple linear regression by municipality between malaria cases and selected variables (insecticide volume, temperature, rainfall and deforestation), from 2019 to 2023

Municipality	Variable	Coef. (β)	Value -p	95%CI bottom	95%CI top	R^2 adjusted
Barcelos	Insecticide (kg)	-0,94	0,21	-2,72	0,84	0,34
	Temperature (°C)	542,1	0,44	-1110	2194	0,27
	Precipitation (mm)	-30,2	0,32	-102,3	41,9	0,35
	Deforestation (km²)	-168,4	0,22	-479,6	142,7	0,43
Tefé	Insecticide (kg)	-0,15	0,67	-1,18	0,88	0,04
	Temperature (°C)	634,5	0,29	-702,2	1971,2	0,36
	Precipitation (mm)	-11,7	0,57	-59,2	35,9	0,12
	Deforestation (km²)	78,3	0,42	-153,4	310,0	0,29
São Gabriel da Cachoeira	Insecticide (kg)	0,66	0,28	-0,76	2,08	0,27
	Temperature (°C)	-542,0	0,51	-2021	910,1	0,23
	Precipitation (mm)	26,1	0,40	-52,3	104,6	0,31
	Deforestation (km²)	322,0	0,37	-546,3	1190	0,33

CI: confidence interval.

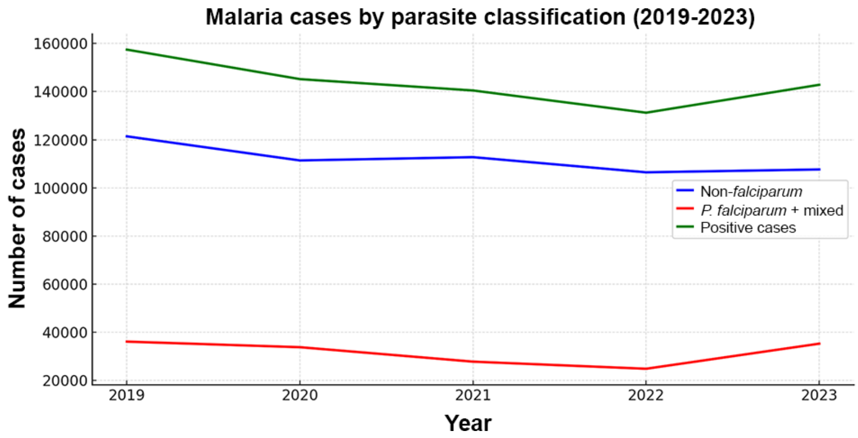

Fig. 1: autochthonous malaria cases in Brazil by area of infection, 2019 and 2023. Source: Sivep-Malária.

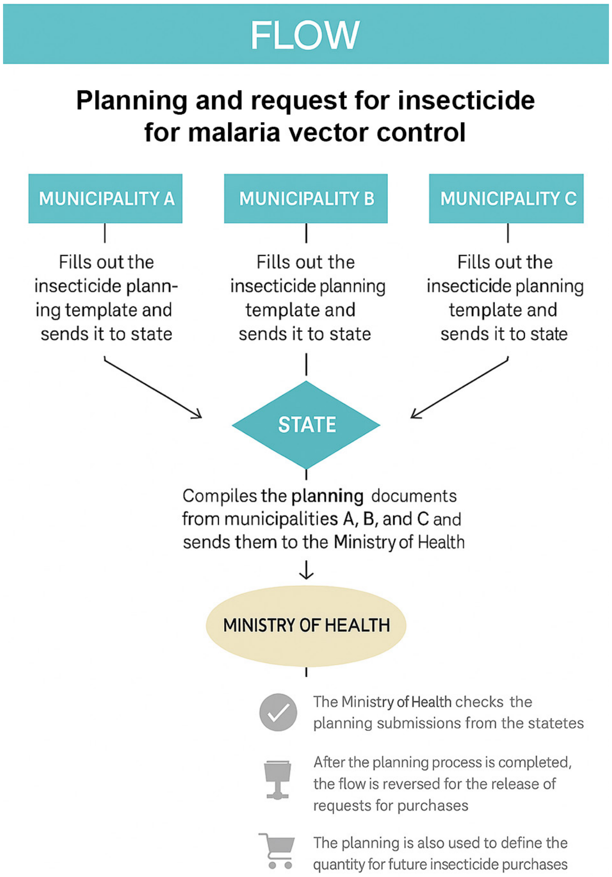

Fig. 2: current flowchart of the National Program for Malaria Prevention, Control, and Elimination's insecticide chain of custody.

Programação de Inseticidas para Malária

DSEI ______ **Atender período:** ______ **a** ______ **Número de casos:** ______

Número de bombas: ______ **Número de agentes p/ BRI:** ______ **Número de ciclos previstos:** ______

	Existentes	
	Urbana	Rural
Nº de Pólos/Postos	0	
Casas/Ocas/Malocas	0	
População	0	

	BRI Executado	
	Urbana	Rural
Nº de Pólos/Postos	0	
Casas/Ocas/Malocas	0	
População	0	

	Planejamento BRI	
	Urbana	Rural
Nº de Pólos/Postos	0	
Casas/Ocas/Malocas	0	
População	0	

Inseticida	Quantidade de cargas necessárias	Estoque Estratégico (+5%)	Estoque existente no DSEI	Quantidade solicitada	Capacidade de operação	Quantidade de cargas por caixa	TOTAL
A	B = (ocas/malocas) x ciclos	C = B x 1,05	D	E = C-D	F	G	H
Etofenprox PM 20%	0	0	20	-20	0	40	0

Fig. 3: model spreadsheet for malaria insecticide programming.

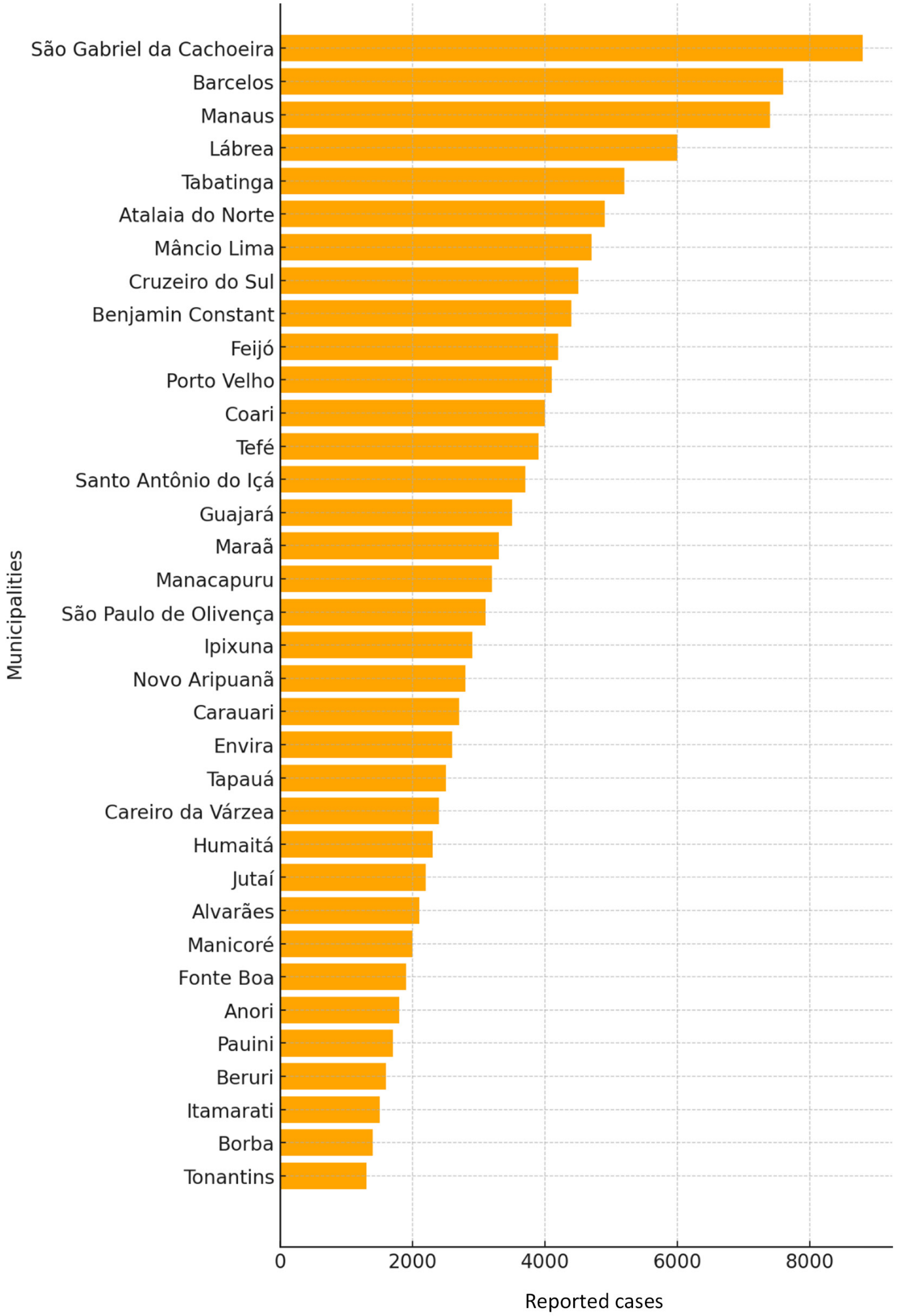

Fig. 4: list of the 35 municipalities with the highest national malaria burden, year 2022. Malaria cases according to municipality of notification and year cure verification slides (CVS) excluded. *Plasmodium vivax* includes *P. vivax* infections and results of the non-*falciparum* rapid diagnostic test. Mixed malaria includes cases of co-infection with *P. falciparum* + *P. vivax* and *P. falciparum* + *P. malariae*. Source: Epidemiological Surveillance Information System (Sivep-Malaria), Notifiable Diseases Information System - Sinan/SVSA/MS.

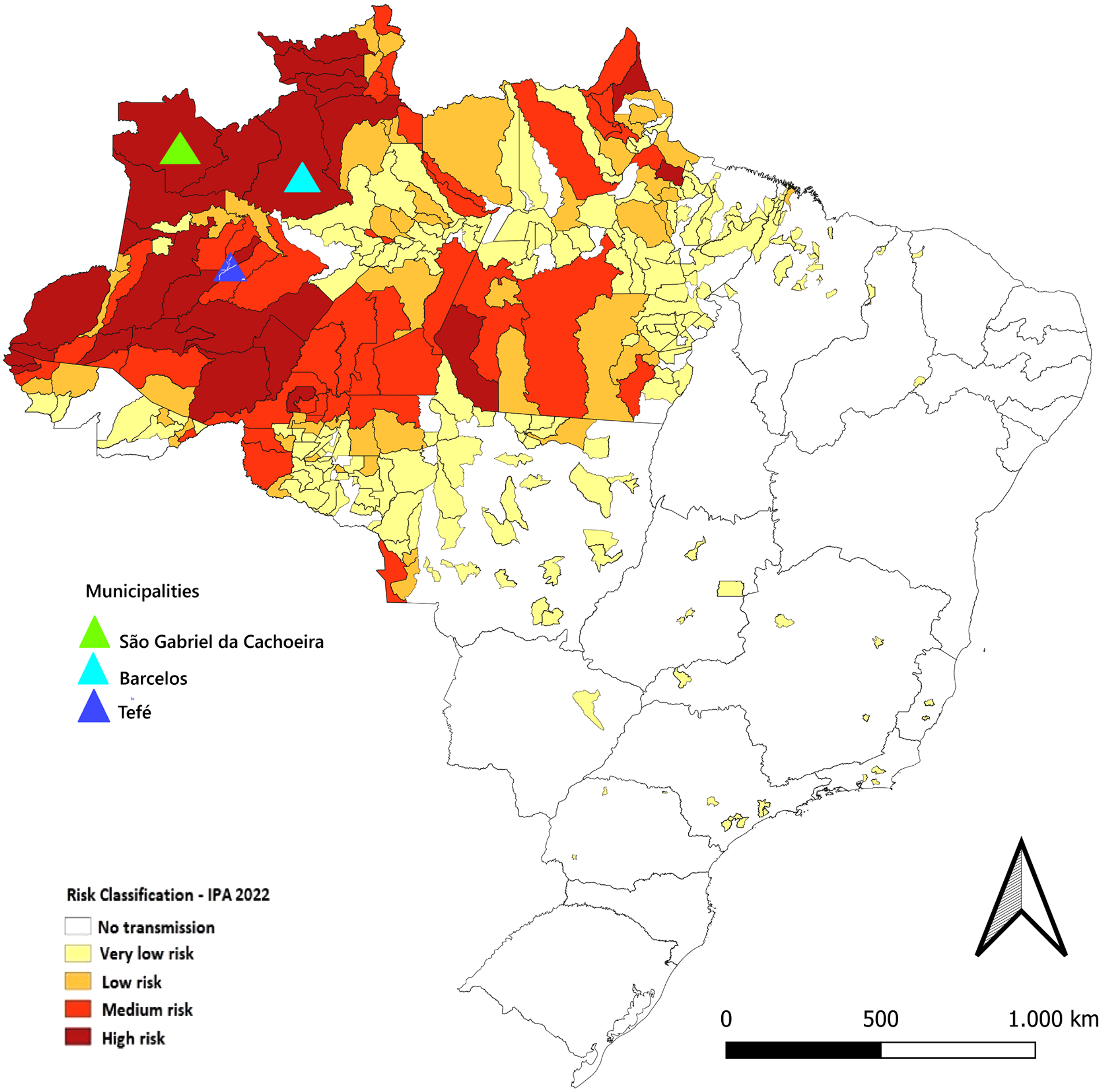

Fig. 5: malaria risk map by municipality of infection, highlighting the study municipalities, Brazil, 2022 - Source: Epidemiological Surveillance Information System (Sivep-Malaria), Ministry of Health (2022).

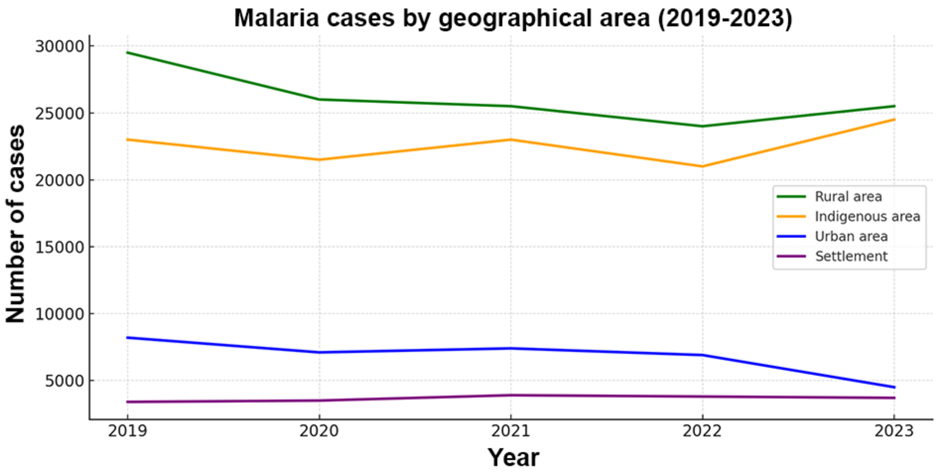

Fig. 6: autochthonous malaria cases in the state of Amazonas by area of infection, 2019 and 2023. Source: Epidemiological Surveillance Information System (Sivep-Malaria).

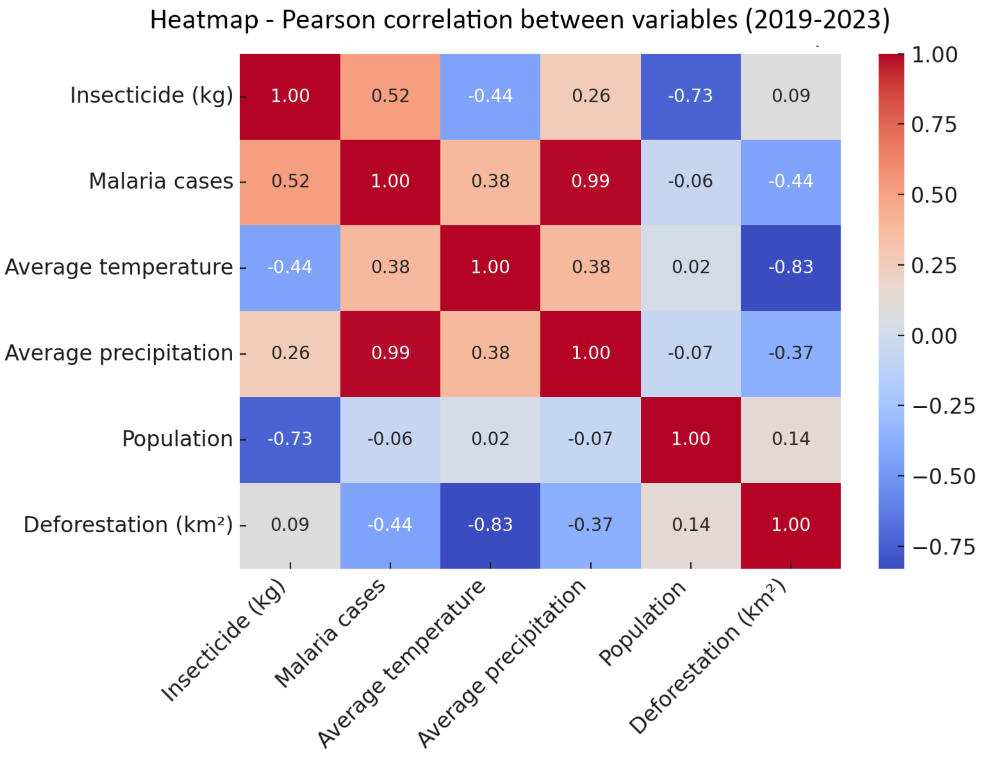

Fig. 7: Pearson correlation heat map, climate variables, malaria cases, insecticide dispensation, population base and deforestation from 2019 to 2023.

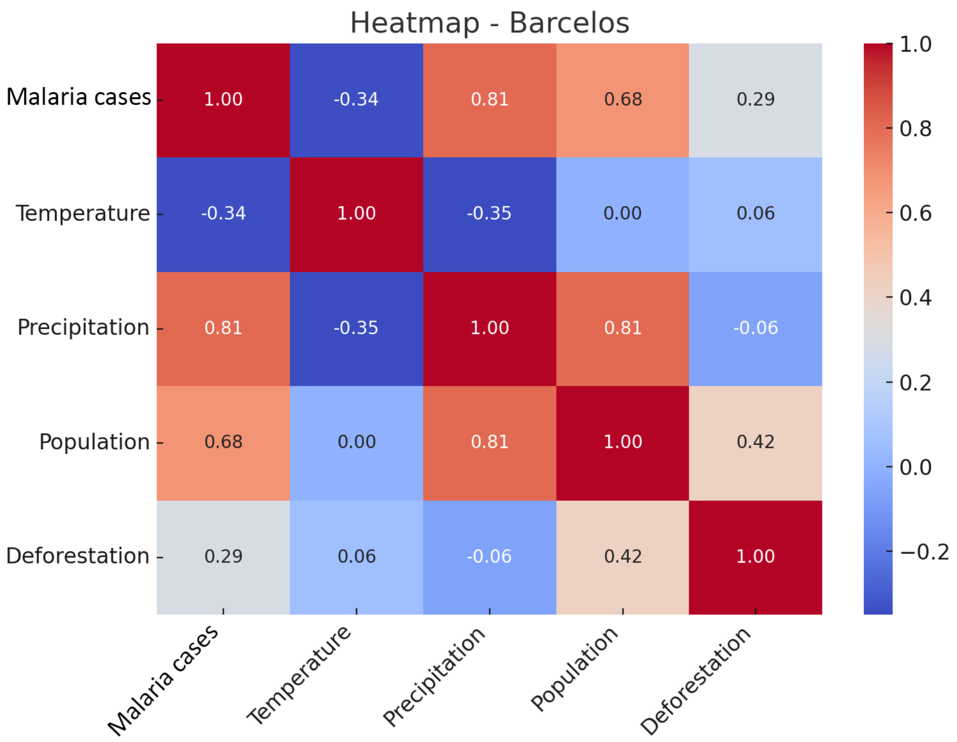

Fig. 8: Pearson correlation heat map, climate variables, malaria cases, insecticide dispensation, population base and deforestation in the municipality of Barcelos, from 2019 to 2023.

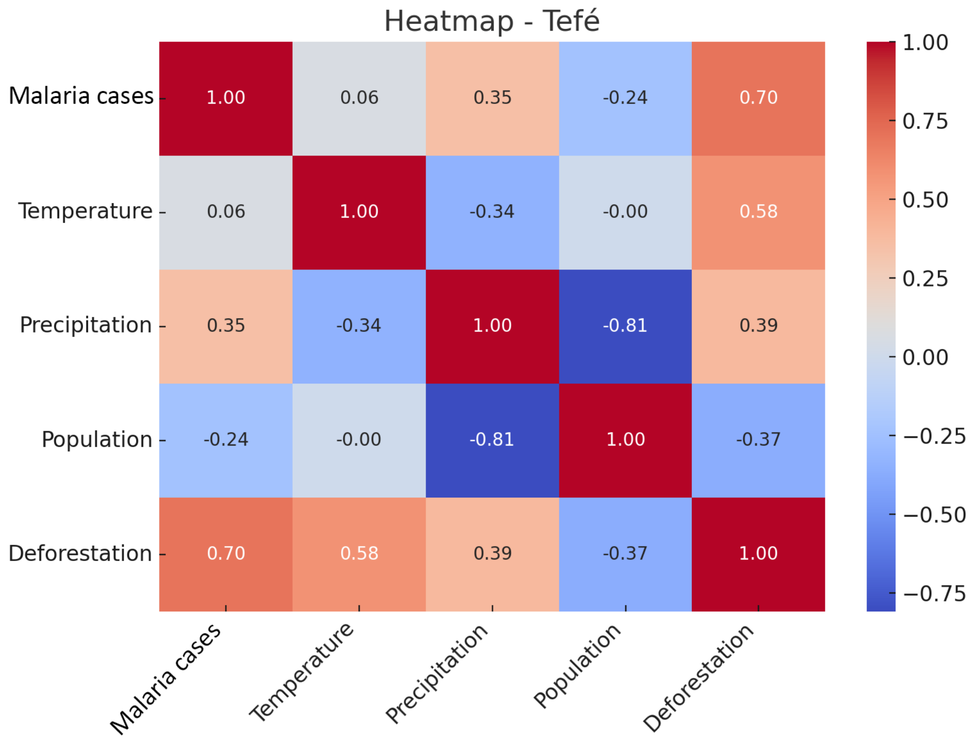

Fig. 9: Pearson correlation matrix, climate variables, malaria cases, insecticide dispensation, population base and deforestation in the municipality of Tefé, 2019 to 2023.

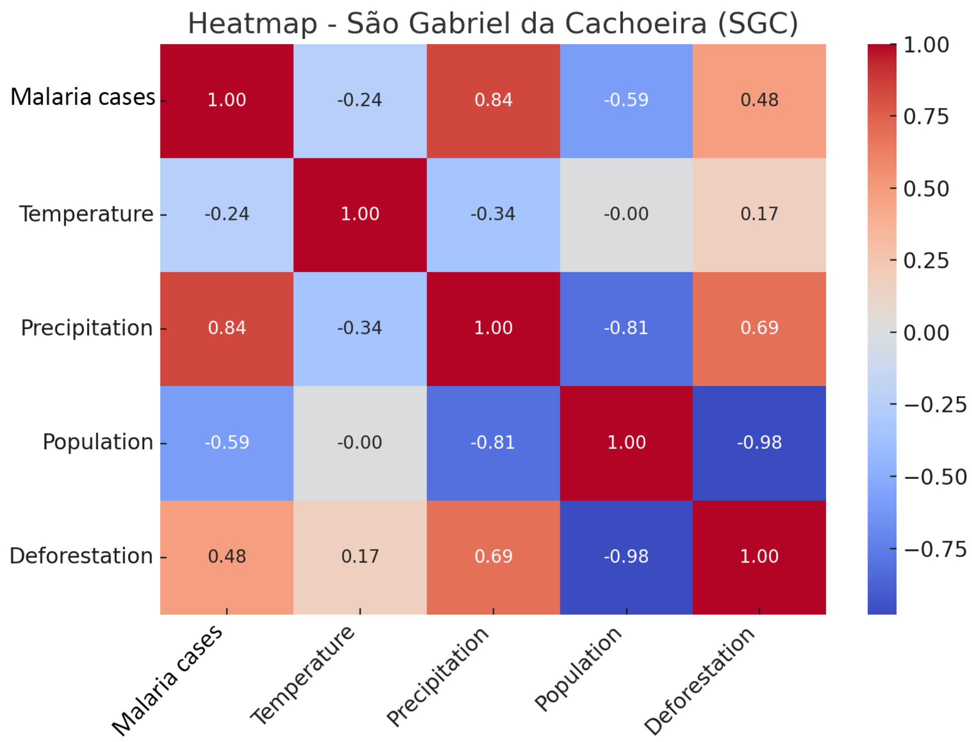

Fig. 10: Pearson correlation heat map, climate variables, malaria cases, insecticide dispensation, population base and deforestation in the municipality of São Gabriel da Cachoeira, 2019 to 2023.

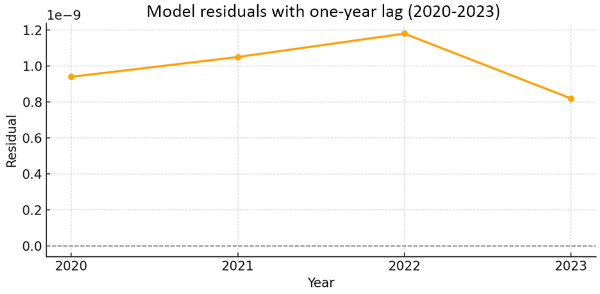

Fig. 11: residuals graph of the multiple linear regression model with a time lag (1-year lag), considering the amount of insecticide distributed in the previous year as the predictor variable.

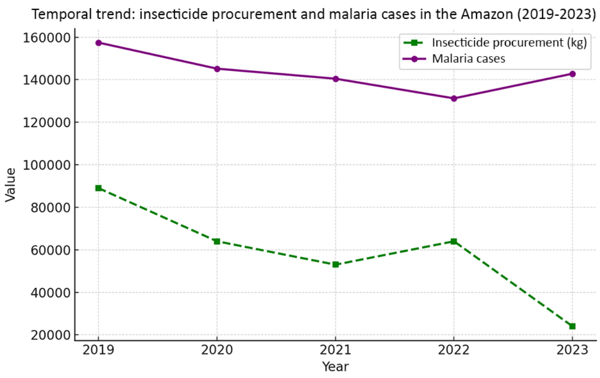

Fig. 12: historical series of reported malaria cases and dispensation in kilograms of Etofenproxi PM sc 20% insecticides, from 2019 to 2023 (LVC excluded) and historical series of insecticide purchases in the Amazon. Source: Epidemiological Surveillance Information System (Sivep-Malaria) and Strategic Inputs Information System - SIES/MS.

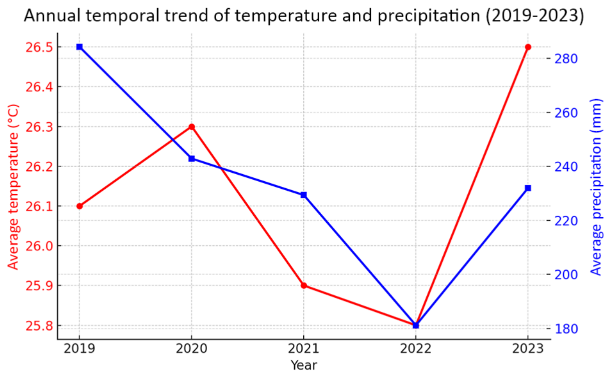

Fig. 13: annual temporal trend of temperature and precipitation in the Amazon region from 2019 to 2023. Source: National Institute of Meteorology (INMET).

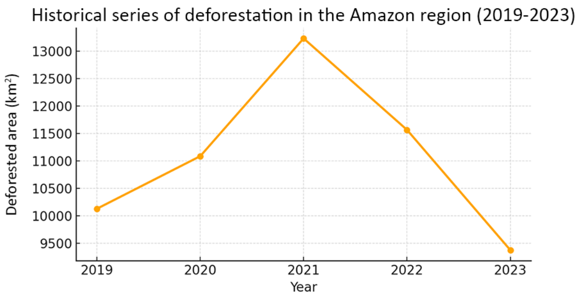

Fig. 14: historical series of deforestation in the Amazon region from 2019 to 2023. Source: National Institute for Space Research (INPE), Programa de Monitoramento da Floresta Amazônica Brasileira - PRODES Project.
